# Modifying the Electrocatalyst–Ionomer Interface via Sulfonated Poly(ionic liquid) Block Copolymers to Enable High-Performance Polymer Electrolyte Fuel Cells

**DOI:** 10.1021/acsenergylett.0c00532

**Published:** 2020-04-29

**Authors:** Yawei Li, Tim Van Cleve, Rui Sun, Ramchandra Gawas, Guanxiong Wang, Maureen Tang, Yossef A. Elabd, Joshua Snyder, K. C. Neyerlin

**Affiliations:** † Chemistry and Nanoscience Center, National Renewable Energy Laboratory, Golden, Colorado 80401, United States; ‡ Department of Chemical Engineering, Texas A&M University, College Station, Texas 77843, United States; § Department of Chemical and Biological Engineering, Drexel University, Philadelphia, Pennsylvania 19104, United States

## Abstract

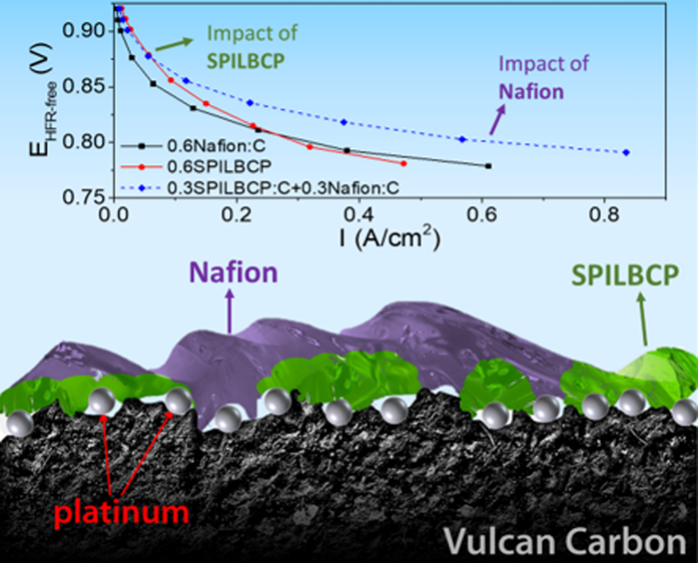

Polymer electrolyte membrane fuel cell (PEMFC) electrodes with a 0.07 mg_Pt_ cm^–2^ Pt/Vulcan electrocatalyst loading, containing only a sulfonated poly(ionic liquid) block copolymer (SPILBCP) ionomer, were fabricated and achieved a ca. 2× enhancement of kinetic performance through the suppression of Pt surface oxidation. However, SPILBCP electrodes lost over 70% of their electrochemical active area at 30% RH because of poor ionomer network connectivity. To combat these effects, electrodes made with a mix of Nafion/SPILBCP ionomers were developed. Mixed Nafion/SPILBCP electrodes resulted in a substantial improvement in MEA performance across the kinetic *and* mass transport-limited regions. Notably, this is the first time that specific activity values determined from an MEA were observed to be on par with prior half-cell results for Nafion-free Pt/Vulcan systems. These findings present a prospective strategy to improve the overall performance of MEAs fabricated with surface accessible electrocatalysts, providing a pathway to tailor the local electrocatalyst/ionomer interface.

Polymer electrolyte membrane (PEM) fuel cells have been well-suited for market applications as power supplies for electric vehicles because of their high electrical efficiencies, relatively low operating temperature, and lack of harmful exhaust.^1,2^ To date, however, further commercial viability and greater market penetration is restrained by requisite platinum-group-metal (PGM) loadings which directly affect the cost of PEM fuel cells. Sluggish cathode oxygen reduction reaction (ORR) kinetics remains a major limitation^3^ engendering higher platinum (Pt) content at the cathode.^4^ Over the past decade, vast resources have been devoted to developing low-PGM electrodes.^4,5^ To facilitate high-performing low-PGM cathodes, various strategies have been employed, including the development of Pt alloys,^3,6−9^ optimization of Pt accessibility,^10,11^ and the tailoring of ionomer properties^12−14^ and distribution^15−17^ in the catalyst layer (CL). However, translating observed improvements in ORR performance at the rotating disk electrode (RDE) level to more relevant membrane electrode assembly (MEA) configurations has often proven difficult and is confounded by both the inclusion of ionomers (proton conducting polymers) and the alteration of dispersion and deposition methods.

Although the presence of ionomers, such as perfluorosulfonic acids (PFSAs), is critical for proton transport throughout the electrode, the specific adsorption of ionomer sulfonic acid groups onto the Pt surface has been suggested to poison catalyst active sites, impairing oxygen reduction reaction (ORR) kinetics at the RDE level.^18−20^ This effect was not observed at the MEA level in the more recent work^16^ of Van Cleve et al. In that work, sulfonate adsorption and mass or specific activity showed no direct correlation for a surface accessible electrocatalyst (i.e., Pt on Vulcan carbon (Pt/Vu)). In a more binary sense, Kongkanand and co-workers demonstrated lower sulfonate adsorption for high surface area porous carbon-supported Pt (Pt/HSC) when compared to surface accessible (Pt/Vu) electrocatalysts, eluding to a possible correlation of Pt location within the carbon support, limited ionomer interaction, and improved specific activity.^10^ Because of the anecdotal and nebulous relationship between sulfonate adsorption and Pt kinetics at the MEA level, no mathematical relationship yet exists to describe Pt performance as a function of sulfonate adsorption.

In contrast, the impact of Pt-oxide coverage on ORR kinetic performance has been observed at both RDE and MEA levels^21,22^ with a reported transition in Tafel slope from ca. 60 mV/dec at high potentials (>0.75 V *iR*-free vs RHE) to ca. 120 mV/dec at low potentials (<0.75 V *iR*-free vs RHE).^22−24^ RDE experiments in HClO_4_ have indicated that ORR kinetics is predominantly controlled by the potential-dependent surface coverage of oxygen species.^23^ Additionally, Subramanian et al. studied the impact of Pt-oxide on ORR kinetics under the fuel cell operating conditions, contrasting the use of a simple Tafel kinetics model and a coverage-dependent ORR kinetic model.^22^ At high potentials where measured Pt-oxide coverage increased, Tafel kinetics decreases because of a reduction in the effective active Pt site density and/or an increase in the barrier to bind the ORR intermediates on oxidized Pt sites.^22,23^ Such studies underscore both a critical need to further the understanding of the near-surface electrocatalyst environment, as well as provide hope that modification of the catalyst–electrolyte (catalyst–ionomer) interface can be a gateway to improved ORR kinetics.

Modification of the catalyst–electrolyte (catalyst–ionomer) interface to enhance ORR activities through the integration of ionic liquids (ILs) has gained more interest since it was first reported for nanoporous alloy ORR electrocatalysts.^25^ A range of IL chemistries have been investigated for PGM^26−32^ and PGM-free^33−36^ catalysts, leading to reduced overpotentials and remarkable ORR activity enhancements. While questions remain regarding the mechanism for improved ORR performance, explanations center around increased oxygen solubility,^25,26,30,31^ inhibition of Pt oxidation,^28,29,31^ and lower adsorption of nonreactive species.^27,28,31,32^ Despite promising early work, the practical implementation of ILs in MEAs has been a difficult path. The challenges include controlling IL film thickness to limit additional O_2_ transport resistance^27^ and maintaining the IL in the electrode without removal over the course of testing. In an effort to overcome such challenges, a unique block copolymer was synthesized, where one block contains sulfonic acid moieties and the other contains ionic liquid (methylimidazolium bis(trifluoromethylsulfonyl)imide, [MIm][TFSI]) moieties.^37^ Using a synthesis process similar to earlier work by Meek et al.,^38^ a sulfonated poly(ionic liquid) block copolymer (SPILBCP) was developed, and the chemical structure is provided in the Supporting Information (Figure S1). Such SPILBCP can possess multiple desired properties related to ionic conductivity, O_2_ permeability, and thermal/electrochemical stability.^38^ As with any novel material, the key is translating observed *ex situ* and/or half-cell enhancements to the operando environment. This work focuses on the incorporation of SPILBCPs into functioning PEM fuel cell electrodes. Externally accessible electrocatalysts (Pt/Vu) were used in order to affect the local catalyst–ionomer interface, enhance electrochemical performance, and identify the responsible mechanism.

Panels a and b of Figure 1 show the improvement in both catalyst mass-based activity (*i*_m_^0.9 V^) and ECA-based activity (specific activity – *i*_s_^0.9 V^) determined at 0.9 V *iR*-free. Previous work^39^ has demonstrated improved kinetic performance of Pt/Vu MEAs with the application of low temperature, low voltage, and high RH holds. In this study, these “voltage recovery” (VR) cycles were employed to achieve optimal MEA performance as previously described.^39^ Consistent with earlier results on Pt/Vu electrodes, 2–3 VR cycles were required to reach peak mass activity with Nafion-containing MEAs. However, more VR cycles are required for SPILBCP incorporated samples to achieve peak power, something that may be related to the near-surface optimization of the electrocatalyst–ionomer interface and the additional hydration process within IL-functional group domains required to assist proton conductivity.

**Figure 1:**
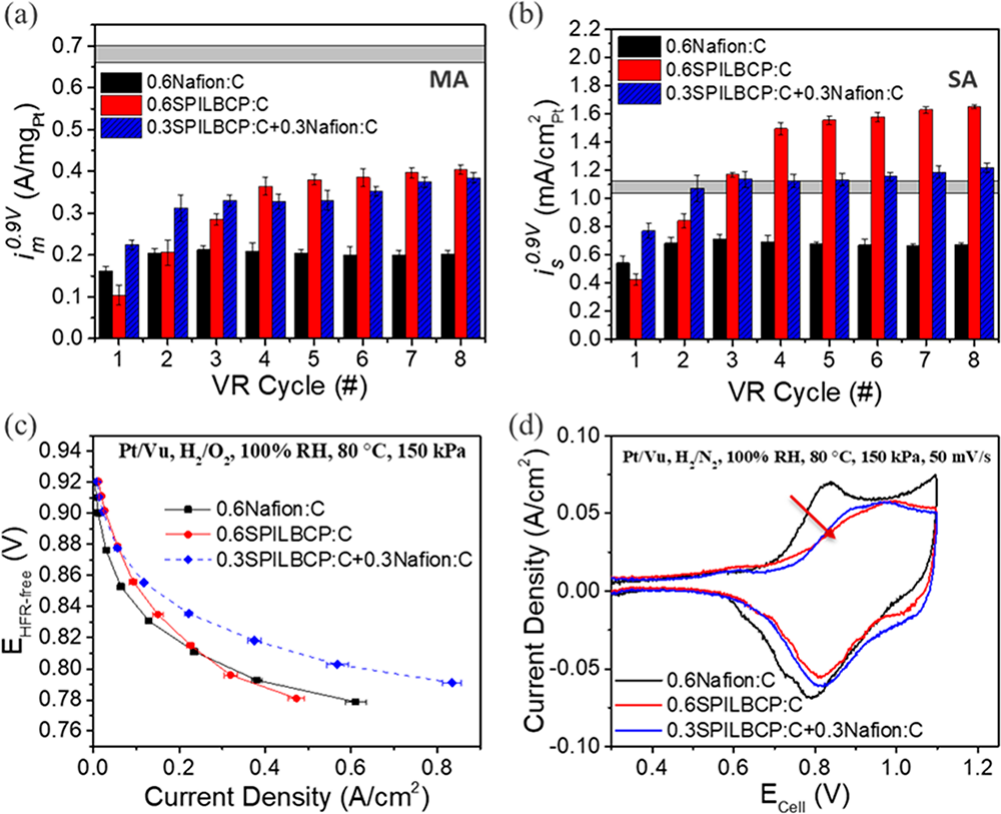
(a) Mass-based (*i*_m_^0.9 V^) and (b) ECA-based (*i*_s_^0.9 V^) performance of fully conditioned Pt/Vu MEAs from H_2_/O_2_ performance at 150 kPa, 80 °C, and 100% RH. Horizontal gray bars represent reported RDE values of mass-activity (MA) and specific-activity (SA) for Nafion-free Pt/Vu systems in ref (19). (c) Average H_2_/O_2_ performance data (80 °C, 100% RH, 150 kPa_abs_ total pressure) at peak performance. (d) Surface oxidation using cyclic voltammetry (80 °C, 100% RH, 150 kPa_abs_ total pressure) at 50 mV/s obtained for low-loading Pt/Vu MEAs (0.07 mg_Pt_ cm^–2^). All error bars correspond to the standard deviation from at least 2 experiments.

Because SPILBCPs are not easily dispersed in alcohol-based solvents (nPA, iPA) commonly used for the catalyst suspensions,^40,41^ and because both the resulting ionomer film^42^ and electrode catalyst–ionomer microstructure are a strong function of ink formulation,^43^ i.e., components and composition,^16^ an acetonitrile-based solvent mixture was used to fabricate the electrodes in this study. Although acetonitrile has been purported to reduce the electrochemical active surface area (ECA) by strongly interacting with Pt sites,^44,45^ no such effect was observed here. Figure S2 shows a negligible difference in hydrogen adsorption/desorption features between electrodes deposited from isopropanol and acetonitrile-based catalyst inks, indicating the removal of adsorbed contaminant species after potential cycling. After some investigation into the effect of ink composition on particle aggregation, dynamic lighting scattering (DLS) revealed that a mass-based ink composition of 60% water and 40% acetonitrile produced a minimum catalyst–ionomer particle aggregation for Pt/Vu electrocatalyst inks containing both Nafion and SPILBCP (Table S1). In these formulations, the average aggregate size (*Z*_avg_) of Pt/Vu was shown to be consistent with previous studies in *n*-propanol/water mixtures (Table S1).^16,43^

Figure 1c summarizes the geometric performance of Pt/Vu catalyst-coated membranes (CCMs) during H_2_/O_2_ polarization experiments at maximum performance after the application of VR cycles (3 for Nafion only, and 8 for the CCMs containing SPILBCPs). Of note, despite the change to an acetonitrile/water ink formulation, the Nafion-based MEAs achieve a mass activity nearly identical to previously reported results for nPA/water-based MEAs at an identical ionomer-to-carbon (I/C) ratio.^16,39,46^ However, when Nafion is replaced with SPILBCP, a significant performance increase was observed in the low current density (LCD) region (>0.8 V_RHE_) resulting in improvements to both *i*_m_^0.9 V^ and *i*_s_^0.9 V^. As shown in Figure 1a,b, SPILBCP-containing CCMs (0.6SPILBCP:C) exhibit *i*_m_^0.9 V^ and *i*_s_^0.9 V^ approximately double those observed for Nafion-only CCMs (0.6Nafion:C). This result is a milestone for the PEMFC community as it is the first time that *i*_s_^0.9 V^ values determined from an MEA were observed to be on par with prior RDE results for Nafion-free Pt/Vu systems (e.g., *i*_s_^0.9 V^ ca. 1.05–1.12 mA/cm^2^_Pt_).^19,47^

Correlating with the increase in *i*_s_^0.9 V^, Figure 1d shows the suppression of surface oxidation for SPILBCP containing samples where the positive shifts in Pt oxidation peak potential (∼0.8 V_cell_) are indicative of decreased coverage of oxide species. The decrease in Pt-oxide coverage was previously shown to increase ORR activity for Pt alloys^3,6−9^ and IL incorporated systems.^26−32^ In fact, while enhancements in specific activity are oftentimes associated with the inhibition of sulfonate anion adsorption, IL functionality has been shown to influence the electrocatalyst–ionomer interface, altering Pt-oxide formation.^27−29^ Prior works^28,30,32^ have indicated that the introduction of hydrophobic IL [BMIm][TFSI] to Pt surfaces may destabilize Pt-bound oxygenated species and impede the formation of an interfacial ice-like water network known to slow ORR kinetics at high operating potentials.^27,28,48,49^ Nevertheless, despite possessing an improved kinetic performance, the geometric performance of the SPILBCP-only samples are overtaken by the Nafion-only samples at current densities above 0.3 A/cm^2^ (Figure 1c), likely because of an increase in electrode proton resistance for the SPILBCP containing samples.

Because H_2_/O_2_ polarization curves yield limited information on proton and, especially, gas transport-related losses, H_2_/air polarization curves were collected at both high and low relative humidity (RH) conditions (panels a and b of Figure 2, respectively). Additionally, to further evaluate the impact of SPILBCP and optimize MEA level performance, various loadings of the block copolymer ionomer were introduced. While the presence of SPILBCP promotes kinetic performance in all cases (Figure S3), neither higher loading (I/C = 0.8) nor lower loading (I/C = 0.3) of the SPILBCP improved the performance in H_2_/air at 100% and 30% RH. Limiting current experiments were performed on MEAs to evaluate the non-Fickian (pressure-independent) O_2_ transport resistance (*R*_nF_), which is inversely related to the high current density (HCD) performance. Figure 2c indicates SPILBCP-only samples have much higher *R*_nF_ in comparison to Nafion-only MEA with similar loading, an effect which is amplified at low RH. Because *R*_nF_ is dependent on Pt active site accessibility, changes in normalized ECA (NECA, so-called “dry proton accessibility”)^10^ of fully conditioned MEAs could help explain the increased *R*_nF_ at lower RHs. Figure 2d indicates that the SPILBCP-only MEAs have much lower NECA (ECA from CO stripping at a given RH relative to total ECA measured at 100% RH) in comparison to Nafion-only MEAs. In fact, SPILBCP samples lose over 70% of their active area at RH = 30%, much higher than the ∼20% ECA loss typically observed in Pt/Vu-Nafion electrodes.

**Figure 2:**
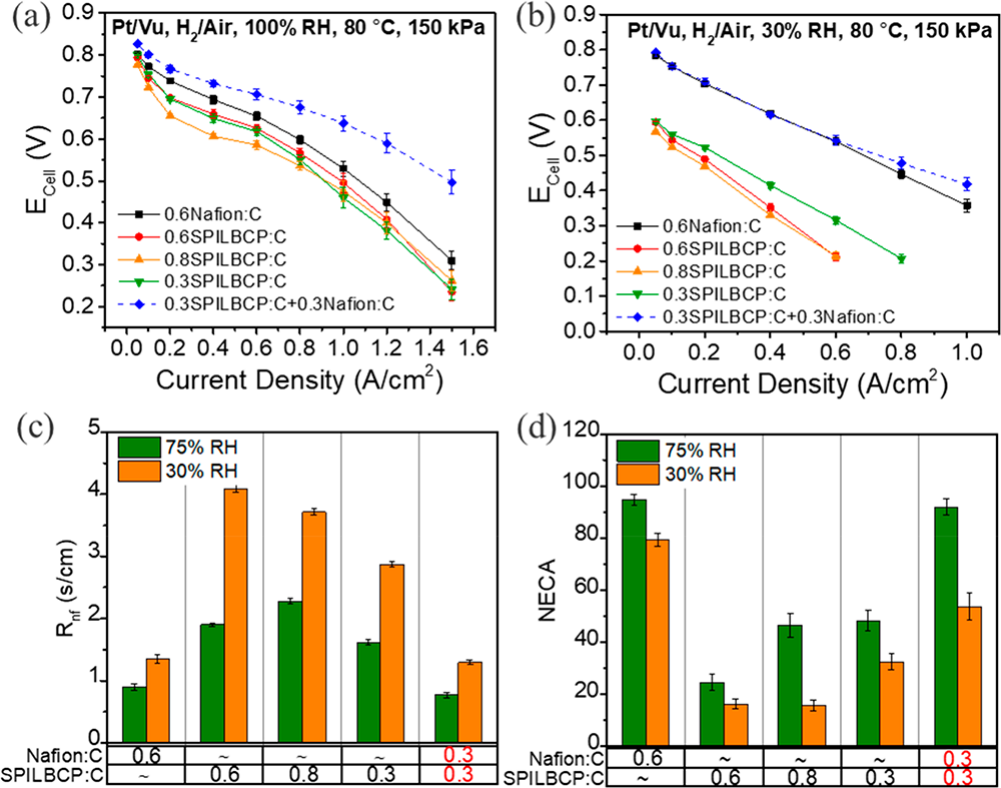
Average H_2_/air performance data at 80 °C, 150 kPa, and (a) 100% RH and (b) 30% RH. (c) Non-Fickian oxygen transport resistances, *R*_nF_, for fully conditioned Pt/Vu MEAs (0.07 mg_Pt_ cm^–2^) determined by limiting current experiments using 5 cm^2^ differential cells. (d) Normalized ECA of fully conditioned Pt/Vu MEAs determined by CO stripping at 80 °C and indicated RH.

Typically, Vulcan-supported Pt catalysts are able to retain the bulk of their ECA regardless of water content because the majority of Pt nanoparticles are located on the exterior of primary carbon particles; however, these primary particles often coalesce into aggregates or larger agglomerates with micro/mesopores within the CL.^50,51^ Unlike Nafion-containing MEAs that maintain facile proton transport, the complex chemical functionality of the SPILBCPs could mean that intramolecular interactions between side chains can limit the formation of well-connected proton pathways throughout the electrode, resulting in an exponential reduction in Pt utilization at low RH conditions. Furthermore, at low RH, the limited water uptake in SPILBCP may condense the ionomer into dry salt where the ionic mobility is significantly inhibited. Thus, at dry conditions, only the electrocatalysts next to a relatively highly conductive Nafion membrane are functioning because of poor ionic conductivity with pure SPILBCP (Figure 3). This appears to be the case in Figure 2b, where the performance of any MEA containing only SPILBCP drops nearly 200 mV at 30% RH H_2_/air when compared to the Nafion containing samples. Because this voltage loss happens nearly at open-circuit voltage, kinetically speaking, it would require 3 orders of magnitude reduction in Pt utilization, a value well beyond that obtained from simple CO stripping measurements in Figure 2d. Consequently, there is a clear need to simultaneous optimize (i) the electrocatalyst–ionomer interface to take advantage of beneficial Pt-oxide suppression, improving electrochemical kinetics, and (ii) the CL microstructure to enable better Pt utilization at low RH, improving mass transport and proton conductivity by forming a highly connected ionomer network.

**Figure 3:**
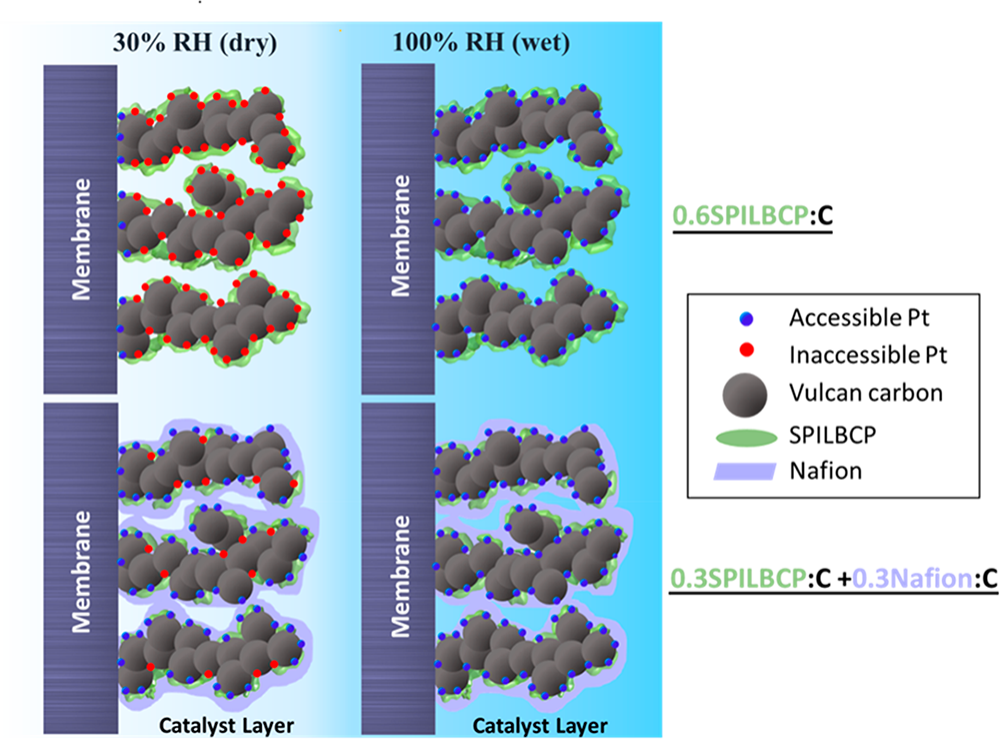
Proposed microstructure of 0.6SPILBCP:C and 0.3SPILBCP:C+0.3Nafion:C electrodes depicting the effect of ionomer distribution on Pt utilization under dry and wet operating conditions.

With the goal of maintaining the enhanced kinetic performance and overcoming the characteristic mass transport challenges of SPILBCP MEAs, both SPILBCP and Nafion ionomers were incorporated into CL to achieve a more optimal electrode structure. According to Figure 2d, MEAs containing both Nafion and SPILBCP (with I/C of 0.3SPILBCP:C+0.3Nafion:C) have significantly higher NECA values, even approaching those of Nafion-only MEAs, which leads to reduced *R*_nF_ values (Figure 2c) and greatly improved performance (Figure 2a,b). This is abundantly clear in Figure 2b, where the OCV of mixed Nafion/SPILBCP MEAs is on par with that of the Nafion-only MEA. At 30% RH, the enhanced kinetic activity for mixed Nafion/SPILBCP MEAs (ca. 2× vs Nafion only, see Figure 1a) is offset by the reduced NECA, netting a similar polarization curve. However, for H_2_/air polarization acquired at 100% RH (Figure 2a), where Pt ECA is nearly identical (Table S2), the improved *i*_m_^0.9 V^ (Figure 1a) and reduced *R*_nF_ (Figure 2c) of the mixed Nafion/SPILBCP electrodes result in a substantial improvement in MEA performance across the kinetic and mass transport-limited regions.

From an electrode structure point of view illustrated in Figure 3, Nafion helps to bridge vacancies between isolated SPILBCP aggregates, forming highly connected ionomer networks and facilitating proton transport in the CL, increasing Pt utilization. This can be further supported by the improved proton conductivity through incorporating both SPILBCP and Nafion into the CL from AC impedance measurements (Figure S4). In addition, the reduced *R*_nF_ values obtained for 0.3SPILBCP:C+0.3Nafion:C compared to 0.6Nafion:C (Figure 2c) can be due to weaker polymer confinement effects with lower loading of Nafion.^52^ As discussed earlier and shown in Figure 1a,b, enhancements to *i*_m_^0.9 V^ and *i*_s_^0.9 V^ are preserved in mixed Nafion/SPILBCP MEAs, consistent with the continued suppression of Pt surface oxidation (Figure 1d). Accounting for metal loading and ECA, oxide coverage was calculated as a function of potential (Figure 4a) from data provided in Figure 1d (details provided in the Supporting Information). These coverages were input into a kinetic model^22^ (eq S2) to predict the specific activity enhancements resulting from oxide suppression on 0.6SPILBCP:C and 0.3SPILBCP:C+0.3Nafion:C electrodes. Figure 4b predicts coverage-dependent ORR kinetics indicating larger Tafel slopes at high potentials for SPILBCP containing MEAs. From the model, both electrodes containing SPILBCP were predicted to show ca. 2× enhancement in kinetic performance at 0.9 V compared to 0.6Nafion:C electrodes, resulting from lower oxide coverage on these electrodes. This is in good argument with 1.8–2.5× enhancements observed experimentally (Figure 1b) and presents a plausible explanation for the kinetic improvement of SPILBCP containing electrodes.

**Figure 4:**
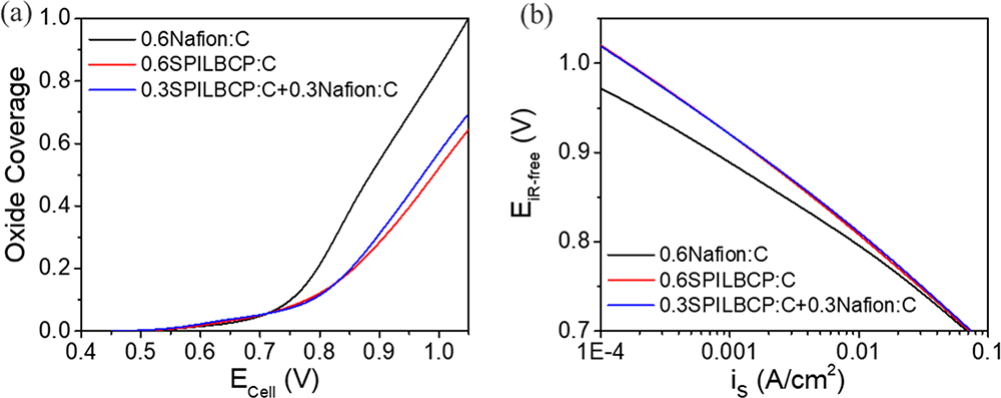
(a) Oxide coverage and (b) coverage-dependent kinetics of Pt/Vu with different ionomers.

In conclusion, a mixed SPILBCP MEA was developed to enable robust operation. MEAs made with mixed Nafion/SPILBCP maintained high NECA at low RH, because of the sulfonated block copolymer, while the inclusion of SPILBCP resulted in a ca. 2× enhancement of *i*_s_^0.9 V^ through reduced Pt-oxide coverage. This modification of the electrocatalyst–ionomer interface enabled *i*_s_^0.9 V^ on par with those from ionomer free RDE experiments. This work represents a promising strategy to improve the overall performance of MEAs fabricated with surface accessible electrocatalysts. These results suggest that future ionomer development should focus on not only intrinsic properties of the ionomer but also how to beneficially incorporate the ionomer(s) within the catalyst micro/nanostructure. Future research will focus on the durability and further optimization of mixed ionomer systems.

## Supporting Information Available

Detailed description of electrode fabrication and assembly, MEA conditioning protocols, *in situ* electrochemical diagnostics (fuel cell performance measurement, CO stripping measurement, O_2_ limiting current experiment, and AC impedance measurement), DLS measurement, and oxide coverage and kinetic model calculations (PDF)
