# Supplementary material for: Modifying the Electrocatalyst–Ionomer Interface via Sulfonated Poly(ionic liquid) Block Copolymers to Enable High-Performance Polymer Electrolyte Fuel Cells
Source: ACS Energy Lett. 2020 Apr 29;5(6):1726–31. doi: 10.1021/acsenergylett.0c00532 (PMC10906942; doi:10.1021/acsenergylett.0c00532)
Supplement: Supplementary file 1 — nz0c00532_si_001.pdf [file nz0c00532_si_001.pdf]

Supporting Information

Modifying the Electrocatalyst-Ionomer Interface via Sulfonated Poly(Ionic Liquid) Block Copolymers to Enable High-Performance Polymer Electrolyte Fuel Cells

Yawei Li[1], Tim Van Cleve[1], Rui Sun[2], Ramchandra Gawas[3], Guanxiong Wang[1], Maureen Tang[3], Yossef A. Elabd[2], Joshua Snyder[3], and K. C. Neyerlin[1*]

[1] *Chemistry and Nanoscience Center, National Renewable Energy Laboratory, Golden, CO 80401, USA*

[2] *Department of Chemical Engineering, Texas A&M University, College Station, TX 77843, USA*

[3] *Department of Chemical and Biological Engineering, Drexel University, Philadelphia, PA 19104, USA*

**Corresponding author: Kenneth.Neyerlin@nrel.gov*

Experimental Methods

Electrode Fabrication and Assembly

All catalyst coated membranes (CCM) were prepared using a similar catalyst ink formulation. Firstly, the ionomer (such as SPILBCP and Nafion D2020 Ion Power) was pre-dispersed in acetonitrile with desired ionomer-to-carbon ratio (I/C) prior to adding water. Next, 47.7 wt% Pt/Vu (TKK, TEC10V50E) was added into a mixture of ionomer, deionized water (DI) and acetonitrile. The catalyst suspensions were then dispersed with 20 s of horn sonication followed by 20 mins of ice-bath sonication.[1,2] The catalyst layer was ultrasonically sprayed onto Nafion NR211 membranes (IonPower) using a Sono-Tek spray station with 25kHz Accumist nozzle at a target catalyst loadings of 0.07 mg_{Pt}/cm^2 for cathodes. Pt loadings on each individual electrode were verified by X-ray Fluorescence Spectroscopy (XRF) (Fisher XDV-SDD). Anode electrodes were prepared with PtCo/HSC (Umicore, Pt30 0670) dispersed in DI:n-PA (70 wt% water) with a I/C of 0.9 (Nafion:C mass ratio) and a 0.03 mg_{Pt}/cm^2 loading.

Once fabricated, the CCMs were assembled into either 50 cm^2 hardware for performance metrics measurements such as H_2/O_2 polarization data, H_2/Air polarization data, kinetic activities, and ECA, or 5 cm^2 differential cells for O_2 limiting current experiments.[3] The CCMs were sandwiched between either two 50 cm^2 SGL 29 BC gas diffusion layers (GDLs) at 25% compression or 5 cm^2 Freudenberg GDLs at 18% compression. The CCMs, GDLs, and polytetrafluoroethylene (PTFE) gaskets were then placed between the flow fields and the bolts tightened to 40 inch-pounds.

In situ Electrochemical Diagnostics

A customized Hydrogenics test station was used to perform all fuel cell testing. All the MEAs tested in this work were subjected to an established conditioning protocol[4] consisting of an initial break-in procedure that activates the cell and a series of voltage recovery steps which were followed by polarization measurements.

Break-in. The break-in procedures begin by heating the cell to 80°C, and holding the cell at an open circuit potential (load equivalent flow rates of 0.8/2.5 = H_2/Air L_{std}/min), followed by a series of 5/10/5 voltage cycles in the fuel cell regime of 0.60 V–0.90 V for 4 minutes.[4]

Voltage Recovery (VR). The voltage recovery (VR) step exposed the cell at 0.1 V_{cell} under H_2/Air (950/500 sccm respectively) for 2 hours at 40 °C and 150% RH.

H_2/O_2 Polarization Curves. The test protocol involved measuring the I-V curves from 0.4 V to OCV at 80 °C at 100 kPa O_2 partial pressure (150 kPa total pressure) and 100 % RH for 4 min per point (average of last 1 min used) in the anodic direction. The ORR mass activities were reported at 0.90 V after applying high frequency resistance (HFR) and hydrogen cross-over corrections.

H_2/Air Polarization Curves. The test protocol involved measuring the I-V curves from 0.3 V to OCV at 80 °C and at 150 kPa total pressure with 75 % RH for 4 min per point (average of last 1 min used) in the anodic direction.

CO Stripping Voltammetry. Pt electrochemical surface area (ECA) was determined by integrating the CO stripping charge (Q_{CO}) obtained from cyclic voltammetry (CV) after the introduction of CO to an equilibrated electrode held at 0.2 V_{cell}. The cathode feed was purged with pure N_2 at 0.25 L_{std}/min prior to the first anodic sweep. CVs were performed immediately at 80 °C and different RH under H_2/N_2 sweeping from 0.05 to 0.9 V at 20 mV/s. 420 μC/cm^2 was assumed as the unit charge for CO integrated areas in determining the ECA.

O_2 Limiting Current Experiments. Limiting current measurements were performed at 80 °C and 75/30% RH, with 0.02, 0.03 and 0.05 mole fraction of oxygen. The limiting current was obtained at total cell pressures of 100, 150, 200 and 300 kPa. Limiting current was measured at constant voltages of 0.30, 0.24, 0.18, 0.12, and 0.06 V and held for 3 minutes. Due to the impact of hydrogen evolution on current densities obtained below 0.1 V, the maximum of the resulting current densities above 0.12 V was reported as the limiting current. Additional details on the procedure were previously described by Baker et. al. [3]

AC impedance measurement. Electrochemical impedance spectroscopy (EIS) experiments were performed on fully conditioned 50 cm^2 MEAs with 0.07 mg_{Pt} cm^{-2} at 80 °C and 100% RH using a Gamry Reference 3000 Potentiostat connected to a Gamry 30k Booster. EIS experiments were measured 50kHz-50mHz at 0.45 V vs RHE with 1 atm H_2 and N_2 at anode and cathode gas lines respectively.

Ink Characterization

Dynamic Light Scattering (DLS). The DLS measurements were performed using Zetasizer Nano ZS (Malvern Instruments Ltd, Malvern, U.K.). These measurements were performed on relatively dilute inks compared to inks used for MEA fabrication at 0.1 wt% Pt/Vu as desired by the technique to avoid multiple scattering. And the DLS measurements of ionomer solutions were carried out at 0.024 wt% using a disposable cuvette cell. At least five measurements were taken to ensure repeatability of the results.

Oxide Coverage and Kinetic Model Calculations

A similar methodology has been described elsewhere.[5] In this study, the anodic sweep of cyclic voltammetry experiments (**Figure 1d**) was used to measure surface oxidization of Pt/Vu electrodes, which occurred between 0.45 and 1.05V_{cell}. All spectra were first corrected by a background current measured at 0.45V to eliminate contributions from H_2 crossover. Slight differences in metal loading and electrochemical surface area were corrected by scaling the background corrected currents accordingly. These corrected spectra were then integrated from 0.45V to each measured potential until the upper bound of 1.05V was reached. These charges were

then normalized by the total oxidization charge observed on the 0.6Nafion:C Pt/Vu electrode between 0.45 and $1.05V_{cell}$, to yield a fractional oxide coverage as a function of cell potential (**Figure 4a**) using Equation S1. The coverage values were directly applied in a model previously developed by Subramanian *et al* (Equation S2) to predict coverage-dependent ORR kinetics displayed in **Figure 4b**.[5]

Note, this simplistic treatment does not account for differences in local O_2 concentration or permeability, which might result from PILBCP integration. Overall, we observe that the mixed ionomer MEAs exhibit better performance than any individual component analogues. However, further improvement will depend on the optimal ratio between two ionomers, to achieve a more appropriate electrode microstructure, and the interaction between the catalyst and PILBCP, which all be the focus of future research.

Oxide Coverage Calculation

$$\Theta_{PtO_x}(V) = \frac{\int_{0.45}^{V} \tilde{i}(V')dV'}{Q_{PtO_x,Nafion}} = \frac{\int_{0.45}^{V} \tilde{i}(V')dV'}{\int_{0.45}^{1.05} \tilde{i}_{Nafion}(V')dV'} \qquad Equation\ S1$$

$\Theta_{PtO_x}, V, and\ \tilde{i},$ represents the oxide coverage, cell voltage, and normalized oxidization current measured on different MEAs, and $Q_{PtO_x,Nafion}$ corresponds to the total oxidization charge observed on the 0.6Nafion:C Pt/Vu electrode evaluated between 0.45 and 1.05V. Figure S5 graphically represents how oxide coverage was calculated.

ORR Kinetics Model Parameters and Calculation[5]

ORR kinetics measured as current density (i) depends many experimental variables such as the operating conditions (O_2 partial pressure p_{o2}, temperature T, cathodic overpotential η), the intrinsic properties of catalytic sites (exchange current density i_0, oxide/adsorbate coverage θ), and parameters related to the reaction mechanism (reaction order w.r.t O_2 γ, charge transfer coefficient α, coverage dependent adsorption energetics ω). R and F correspond to the universal gas constant and Faraday constant, respectively.

$$i = i_0\left(\frac{p_{o2}}{p_{o2,\text{ref}}}\right)^{\gamma}(1-\theta)\exp\left(\frac{-\alpha F\eta}{RT}\right)\exp\left(-\frac{\omega\theta}{RT}\right) \qquad Equation\ S2$$

Parameters	Coverage-dependent ORR model[5]
$\boldsymbol{\alpha}$	0.5
$\boldsymbol{\gamma}$	0.7 ± 0.08
$\boldsymbol{\omega}$, **J**	3.0×10^{3}
$\boldsymbol{i_0}$, $\mathbf{A/cm^2_{Pt}}$	3.0×10^{-5}

Table S1. Z_{avg} diameter of dilute ionomer (0.024 wt%) and catalyst (0.1 wt%)+ionomer (0.024 wt%) dispersions characterized by DLS. The ratios are mass based. w stands by water, and AcN stands by acetonitrile.

	Ink Content (z-avg [nm])				
**Ink Materials**	**5:5-w:AcN**	**6:4-w:AcN**	**7:3-w:AcN**	**8:2-w:AcN**	**9:1-w:AcN**
Nafion only	398.9±75.0	324.5±61.1	453.1±80.7	519.2±56.1	NA/>1000
Pt/Vu+Nafion	271.5±5.1	272.2±4.9	308.0±7.1	284.4±11.9	317.3±9.1
SPILBCP only	52.30±0.3	59.68±0.2	71.25±1.2	167.5±4.3	144.2±6.6
Pt/Vu+SPILBCP	271.6±6.6	252.5±2.7	293.7±7.7	491.3±3.7	573.4±3.7

Table S2. Average electrochemical active surface area (ECA) and roughness factor (RF) for MEA with Pt/Vu with loading of 0.07 mg_{Pt} cm^{-2} after 8 VRs.

**I:C**	**0.6Nafion:C**	**0.6PILBCP:C**	**0.3SPILBCP:C + 0.3Nafion:C**
avg. ECA (m^2 g^{-1}_{pt})	36.2	40.2	39.4
avg. RF (m^2 m^{-2}_{pt})	24.98	27.60	27.55

Table S3. Elemental Composition (wt.%) of SPILBCP

ELEMENT	WT.%
**C**	57.38
**H**	5.20
**N**	4.12
**S**	9.47
**F**	8.44

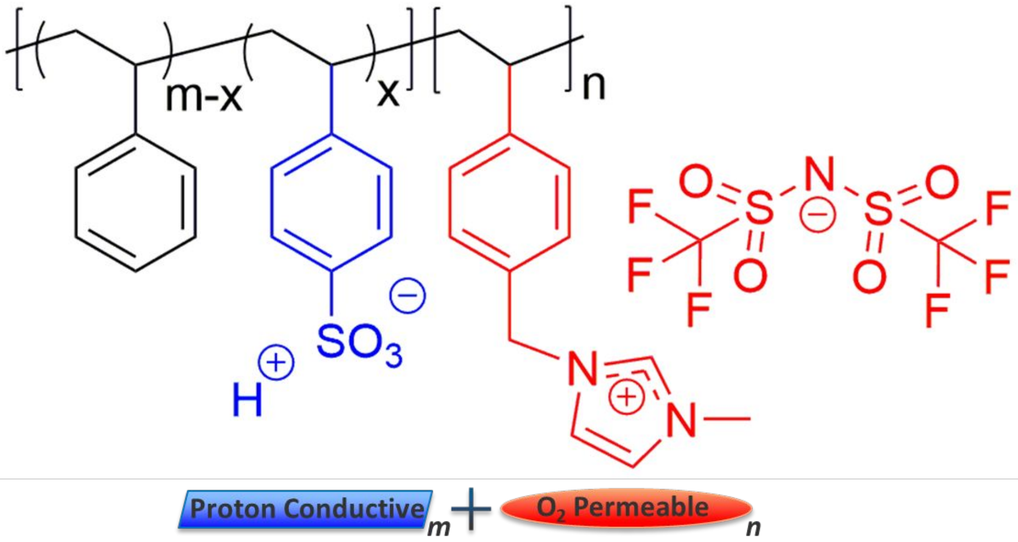

Figure S2. Sulfonated SPILBCP as ionomers for PEM fuel cells.

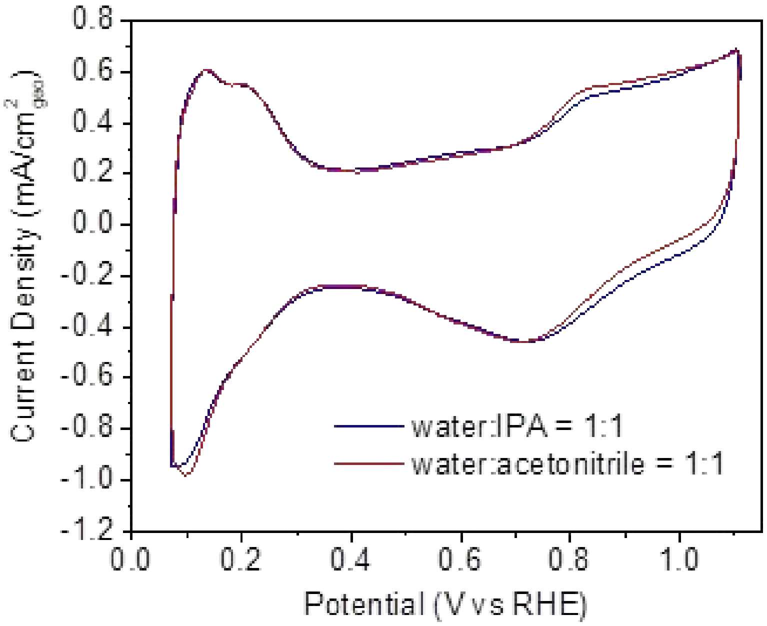

Figure S2. CV scans of Pt/Vu film prepared using ink solvent of water:IPA = 1:1 (*vol*:*vol*) (blue) and water:acetonitrile = 1:1 (*vol*:*vol*) (red) in the fresh electrolyte of 0.1 M $HClO_4$ under Ar at 50 mV/cm^2 after potential cycling of 0.05 ~ 1.2 V vs RHE for 50 cycles.

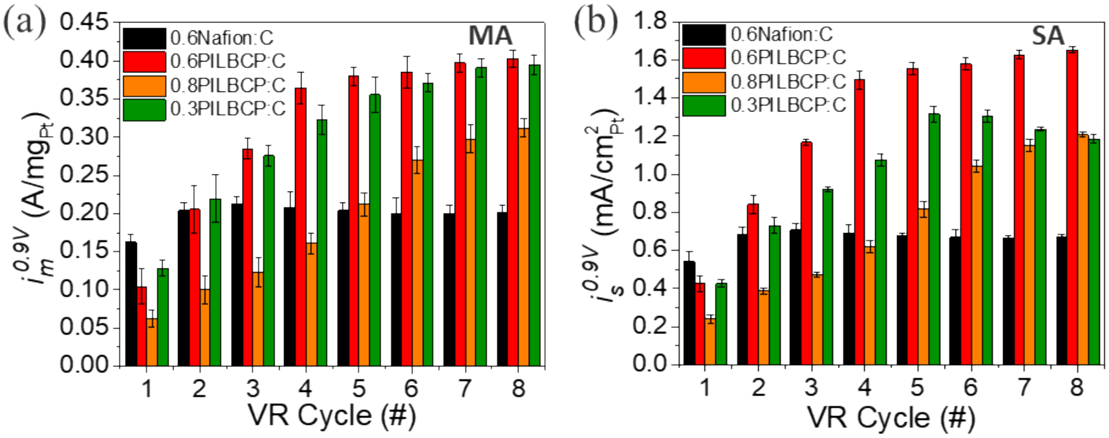

Figure S3. (a) Mass-based ($i_m^{0.9V}$) and (b) ECA-based ($i_s^{0.9V}$) performance of Pt/Vu MEAs from H_2/O_2 performance at 150 kPa, 80 ℃, and 100% RH with indicated ionomer to carbon ratios.

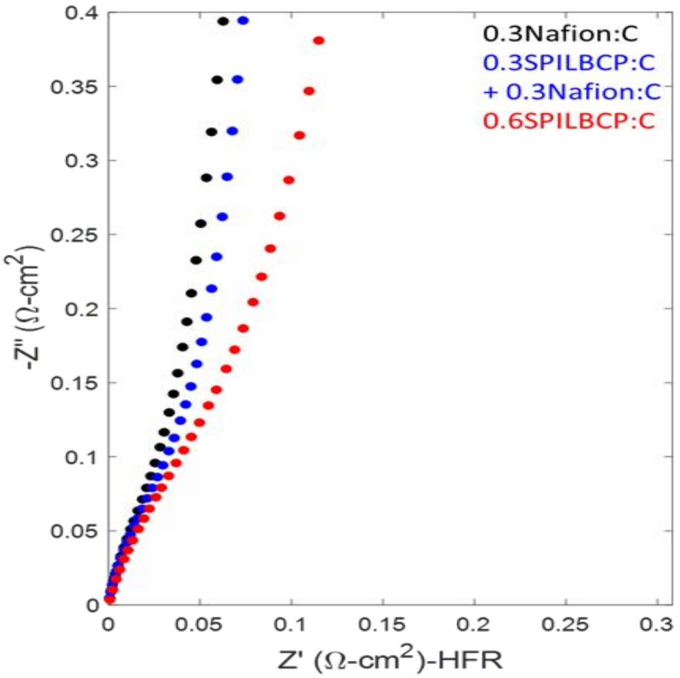

Figure S4 Nyquist plots for fully conditioned Pt/Vu MEAs with indicated ionomer(s) at 80 ℃ and 100% RH.

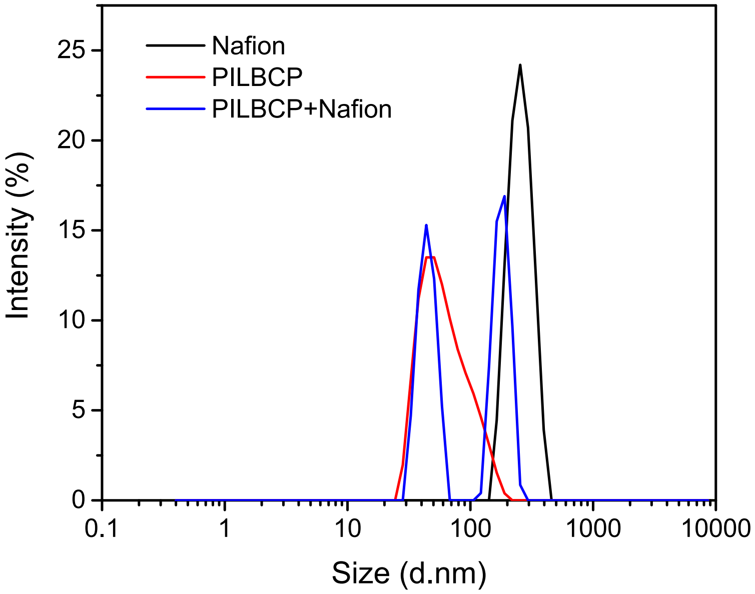

Figure S5. Particle size distribution of dilute (0.024 wt%) ionomer dispersions in the solvent of 60 wt% water and 40 wt% acetonitrile characterized by DLS.

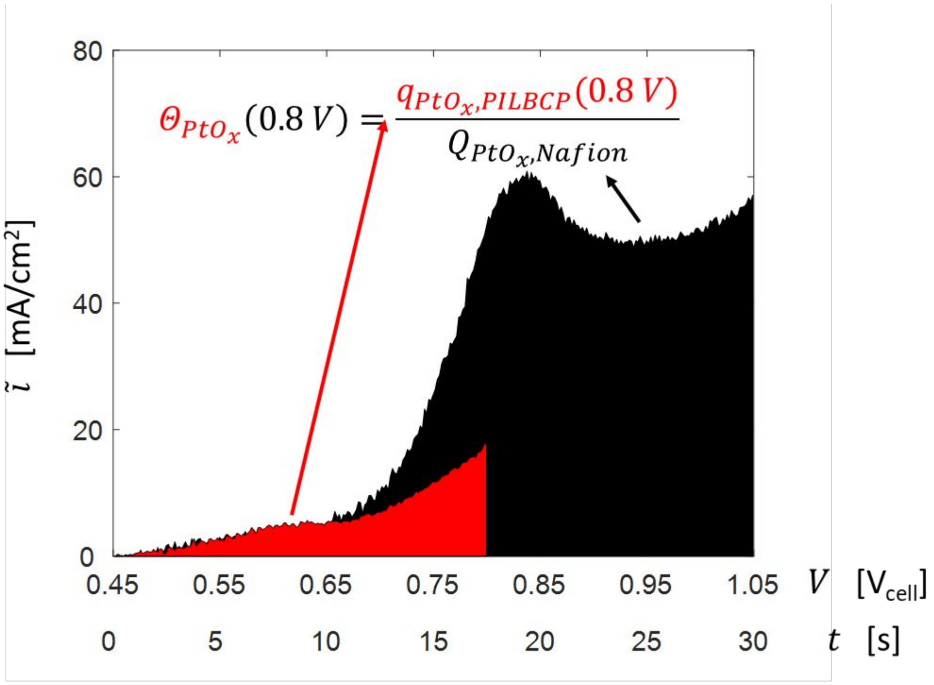

Figure S6. Integration of normalized CV data to calculate oxide coverage on 0.6SPILBCP:C MEA at 0.8Vcell. The red and black regions correspond to the cumulative oxidation charge experience on 0.6SPILBCP:C to 0.80V and 0.6Nafion:C electrodes to 1.05V, respectively.

Reference

1. Wang, M. *et al.* Impact of Catalyst Ink Dispersing Methodology on Fuel Cell Performance Using in-Situ X-ray Scattering. *ACS Appl. Energy Mater.* **2**, 6417–6427 (2019).

2. Van Cleve, T. *et al.* Dictating Pt-Based Electrocatalyst Performance in Polymer Electrolyte Fuel Cells, from Formulation to Application. *ACS Appl. Mater. Interfaces* **11**, 46953–46964 (2019).

3. Baker, D. R., Caulk, D. A., Neyerlin, K. C. & Murphy, M. W. Measurement of oxygen transport resistance in PEM fuel cells by limiting current methods. *J. Electrochem. Soc.* **156**, (2009).

4. Kabir, S. *et al.* Elucidating the Dynamic Nature of Fuel Cell Electrodes as a Function of Conditioning : An Ex-situ Materials Characterization and In-situ Electrochemical Diagnostic Study. *ACS Appl. Mater. Interfaces* **11**, 45016–45030 (2019).

5. Subramanian, N. P., Greszler, T. A., Zhang, J., Gu, W. & Makharia, R. Pt-oxide coverage-dependent oxygen reduction reaction (ORR) kinetics. *J. Electrochem. Soc.* **159**, (2012).
